# Clinical and epidemiological aspects of American cutaneous leishmaniasis with genital involvement^☆^^☆☆^

**DOI:** 10.1016/j.abd.2019.12.010

**Published:** 2020-07-15

**Authors:** Marcelo Rosandiski Lyra, Alan Bittencourt da Silva, Cláudia Maria Valete-Rosalino, Maria Inês Fernandes Pimentel

**Affiliations:** a Laboratory for Clinical Research and Surveillance in Leishmaniasis, Instituto Nacional de Infectologia Evandro Chagas, Fundação Oswaldo Cruz, Rio de Janeiro, RJ, Brazil; b Medical School, Universidade Federal Fluminense, Niterói, RJ, Brazil

**Keywords:** Genital diseases, male, Leishmania braziliensis, Leishmaniasis, cutaneous, Leishmaniasis, mucocutaneous

## Abstract

Genital lesions are an unusual presentation of American cutaneous leishmaniasis. Conditions such as disseminated cutaneous leishmaniasis and HIV infection may be associated with genital involvement. The authors present five cases of American cutaneous leishmaniasis with genital lesions and discuss the clinical and epidemiological aspects observed in this case series.

## Introduction

American cutaneous leishmaniasis (ACL) is an infectious disease caused by protozoa of the genus *Leishmania* transmitted by the bite of infected female sandflies, insects of the genus *Lutzomyia*.^1^, ^2^ Clinically, ACL is divided into localized cutaneous leishmaniasis, disseminated cutaneous leishmaniasis (DL), diffuse cutaneous leishmaniasis, and mucosal leishmaniasis.^1^, ^2^, ^3^

DL constitutes up to 2% of ACL cases and probably occurs due to the lymphatic or hematic spread of the parasite from the bite site.^1^ This clinical form is characterized by the presence of numerous skin lesions, ten or more, distributed in two or more non-contiguous body segments.^3^ Skin lesions are polymorphic and typically consist of acneiform papules, infiltrated or ulcerated plaques, warty lesions and ulcers with a granular bottom and raised edges.^1^, ^3^ Verrucous and vegetating lesions are rare.^3^ Systemic symptoms such as fever, myalgia, asthenia, and weight loss occur in 50% to 75% of cases; mucosal involvement, predominantly in the nasal mucosa, is observed in up to 53% of DL cases.^3^, ^4^

Genital lesions are an unusual presentation of ACL and suggest hematic dissemination in patients with DL or direct inoculation of the parasite in patients with isolated genital lesions who sleep naked outdoors or perform bodily functions in endemic areas of ACL without sanitary facilities.^5^, ^6^, ^7^ Of HIV patients with ACL, 60% presented DL and 27%, genital lesions^8^

## Case reports

**Figure 1:**
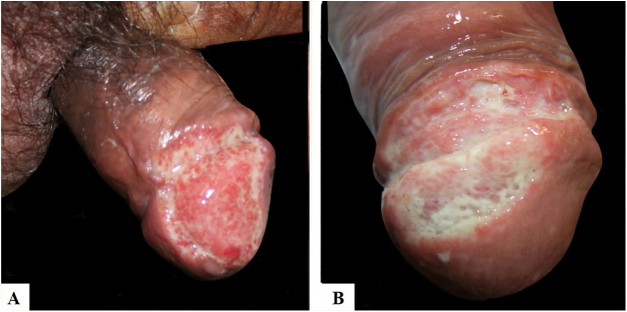
Presence of painless ulceration in the glans and balanoprepucial groove of patients 2 (A) and 4 (B).

**Figure 2:**
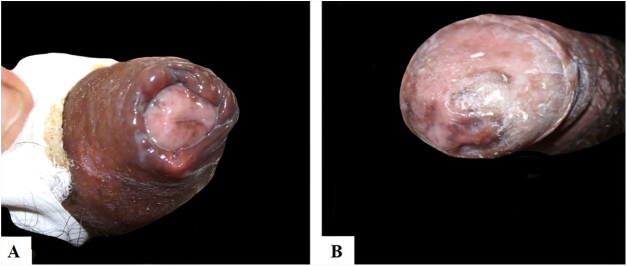
(A) Patient 3, with multiple painful ulcers distributed on the foreskin and body of the penis. (B) Presence of hyperchromic scars on the glans after treatment.

**Figure 3:**
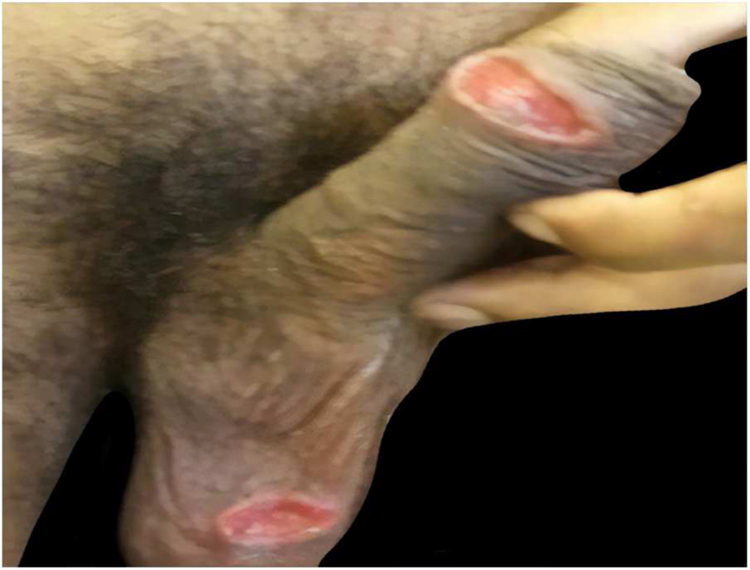
Patient 5, with painless ulcerated lesions in the scrotum and body of the penis.

Table 1 describes the five patients with ACL with genital lesions treated between 2007 and 2019, who comprised the entire series of ACL with genital involvement observed in this institution during this period. The mean age of the patients was 43 years. Among those with DL, a large number of skin lesions were observed, with a mean of 51 lesions. The mean time from the onset of genital lesions until diagnosis was 5.6 months. The diagnosis was confirmed by finding the parasites in one or more of the following tests: direct examination (imprint or scraping), histopathological examination, culture, and/or polymerase chain reaction (PCR) performed in biopsies of the skin lesions. All patients presented upper airway and digestive tract (UADT) mucosal involvement and were tested for HIV and syphilis in order to rule out co-infection. Four of these patients had DL and two were HIV-positive. The glans was the most affected site (Fig. 1). Four patients had painless penile ulcers, except for patient 3, who had penile edema and multiple painful lesions on the foreskin that prevented the exposure of the glans. After treatment, foreskin retraction allowed observation of the lesions on the glans (Fig. 2). Patient five (Fig. 3) presented an ulceration in the body of the penis and another in the scrotum.

**Table 1:** Clinical and epidemiological profile of patients with ACL with genital involvement.

Patient	1	2	3	4	5
Age in years	41	47	65	43	27
HIV	–	–	–	+	+
VDRL	–	–	–	–	–
Number of skin lesions	54	44	55	3	52
Presence of mucosal lesions in UADT	Nasal cavity, oropharynx	Nasal cavity, nasopharynx	Nasal cavity, oropharynx and larynx	Oropharynx, nasopharynx and larynx	Oral cavity
Evolution time until diagnosis	3 months	4 months	6 months	6 months	9 months
Residence (City)	Rio de Janeiro	Saquarema	Rio de Janeiro	Rio de Janeiro	Rio de Janeiro
Treatment	Meglumine antimoniate	Meglumine antimoniate	Liposomal amphotericin B	Liposomal amphotericin B	Meglumine antimoniate

## Discussion

Although Sexually Transmitted Infections (STIs) are the main causes of penile ulcers, other conditions such as fixed drug eruption erythema, autoimmune bullous dermatoses, psoriasis, Behçet's disease, Reiter's syndrome, pyoderma gangrenosum, lichen planus, and squamous cell carcinoma can also cause genital ulcers.^9^ ACL lesions are usually located in exposed areas of the body, and genital involvement is rarely observed.^5^, ^6^, ^7^, ^8^, ^9^, ^10^ Penile lesions in ACL are usually described as painless ulcers with raised edges and insidious evolution, but extensive necrotic ulceration and keratotic plaques have also been reported.^5^, ^6^, ^7^, ^8^, ^10^ Despite the small number of patients in this series, it was observed that the following factors may be associated with genital involvement: mucosal lesions in UADT (100%); DL (80%), especially in cases with a large number of skin lesions; and infection by HIV (40%). The most likely etiological agent in this series was *Leishmania (Viannia) braziliensiss*, as all patients were inhabitants of the state of Rio de Janeiro with no recent history of travel.^1^

ACL should be considered in the differential diagnosis of chronic genital lesions in patients who reside in or travel from endemic areas, especially when associated with mucosal lesions in UADT and multiple, polymorphic skin lesions. Furthermore, the presence of genital lesions can aid in the differential diagnosis of granulomatous diseases with similar clinical presentation, such as paracoccidioidomycosis, histoplasmosis, and disseminated sporotrichosis.

## Final considerations

Genital involvement in ACL probably occurs due to hematic dissemination in patients with DL. Therefore, ACL should be included in the differential diagnosis for patients from endemic areas with genital ulcers, especially in the presence of mucosal lesions in UADT and multiple skin lesions.

## Financial support

Instituto Nacional de Infectologia Evandro Chagas (INI).

## Authors’ contributions

Marcelo Rosandiski Lyra: Approval of the final version of the manuscript; conception and planning of the study; elaboration and writing of the manuscript; obtaining, analyzing, and interpreting the data; intellectual participation in propaedeutic and/or therapeutic conduct of studied cases; critical review of the literature; critical review of the manuscript.

Alan Bittencourt da Silva: Elaboration and writing of the manuscript; obtaining, analyzing, and interpreting the data; critical review of the literature; critical review of the manuscript

Cláudia Maria Valete-Rosalino: Approval of the final version of the manuscript; elaboration and writing of the manuscript; intellectual participation in propaedeutic and/or therapeutic conduct of studied cases; critical review of the literature; critical review of the manuscript.

Maria Inês Fernandes Pimentel: Approval of the final version of the manuscript; elaboration and writing of the manuscript; intellectual participation in propaedeutic and/or therapeutic conduct of studied cases; critical review of the literature; critical review of the manuscript.

## Conflicts of interest

None declared.

## References

[1] Ministério da Saúde (2017). Secretaria de Vigilância em Saúde. Manual de vigilância da leishmaniose tegumentar.

[2] Anversa L., Tiburcio M.G.S., Rochini-Pereira V.B., Ramirez L.E. (2018). Human leishmaniasis in Brazil: A general review. Rev Assoc Med Bras..

[3] Machado G.U., Prates F.V., Machado P.R.L. (2019). Disseminated leishmaniasis: clinical, pathogenic, and therapeutic aspects. An Bras Dermatol..

[4] Rosa M.E.A., Machado P.R.L. (2011). Disseminated leishmaniasis: clinical, immunological, and therapeutic aspects. Drug Dev Res..

[5] Cabello I., Caraballo A., Millán Y. (2002). Leishmaniasis in the genital area. Rev Inst Med Trop Sao Paulo..

[6] Schubach A., Cuzzi-Maya T., Gonçalves-Costa C.S., Pirmez C., Oliveira-Neto M.P. (1998). Leishmaniasis of glans penis. J Eur Acad Dermatol Venereol..

[7] Osório R.C., Barbosa D., Martins M., Leal R., Nascimento D., Fernandes E. (2009). Tegumentary leishmaniasis (TL) caused by Leishmania Viannia braziliensis in genital organs. Gaz Med Bahia..

[8] Lindoso J.A., Barbosa R.N., Posada-Vergara M.P., Duarte M.I., Oyafuso L.K., Amato V.S. (2009). Unusual manifestations of tegumentary leishmaniasis in AIDS patients from the New World. Br J Dermatol..

[9] Yesilova Y., Turan E., Sürücü H.A., Kocarslan S., Tanrikulu O., Eroglu N. (2014). Ulcerative penile leishmaniasis in a child. Indian J Dermatol Venereol Leprol..

[10] Gülüm M., Yeşilovaş Y., Savaş M., Çiftçi H., Yeni E. (2013). A case of giant hyperkeratotic cutaneous leishmaniasis in the penis. Turkiye Parazitol Derg..

